# Recombinant Proteins for Assembling as Nano- and Micro-Scale Materials for Drug Delivery: A Host Comparative Overview

**DOI:** 10.3390/pharmaceutics15041197

**Published:** 2023-04-09

**Authors:** José Luis Corchero, Marianna T. P. Favaro, Merce Márquez-Martínez, Jara Lascorz, Carlos Martínez-Torró, Julieta M. Sánchez, Hèctor López-Laguna, Luís Carlos de Souza Ferreira, Esther Vázquez, Neus Ferrer-Miralles, Antonio Villaverde, Eloi Parladé

**Affiliations:** 1 CIBER de Bioingeniería, Biomateriales y Nanomedicina (CIBER-BBN, ISCIII), Universitat Autònoma de Barcelona, 08193 Bellaterra, Spain; jlcorchero@ciber-bbn.es (J.L.C.);; 2 Institut de Biotecnologia i de Biomedicina, Universitat Autònoma de Barcelona, 08193 Bellaterra, Spain; favaro.mtp@gmail.com; 3 Instituto de Ciências Biomédicas, Universidade de São Paulo, São Paulo 05508-000, Brazil; 4 Departamento de Química, Cátedra de Química Biológica, Facultad de Ciencias Exactas, Físicas y Naturales, ICTA, Universidad Nacional de Córdoba, Av. Vélez Sársfield 1611, Córdoba 5016, Argentina; 5 Instituto de Investigaciones Biológicas y Tecnológicas (IIByT), CONICET-Universidad Nacional de Córdoba, Córdoba 5016, Argentina; 6 Departament de Genètica i de Microbiologia, Universitat Autònoma de Barcelona, 08193 Bellaterra, Spain

**Keywords:** recombinant proteins, protein materials, cell factory, nanoparticles, microparticles, building blocks, biomimetics, protein secretion

## Abstract

By following simple protein engineering steps, recombinant proteins with promising applications in the field of drug delivery can be assembled in the form of functional materials of increasing complexity, either as nanoparticles or nanoparticle-leaking secretory microparticles. Among the suitable strategies for protein assembly, the use of histidine-rich tags in combination with coordinating divalent cations allows the construction of both categories of material out of pure polypeptide samples. Such molecular crosslinking results in chemically homogeneous protein particles with a defined composition, a fact that offers soft regulatory routes towards clinical applications for nanostructured protein-only drugs or for protein-based drug vehicles. Successes in the fabrication and final performance of these materials are expected, irrespective of the protein source. However, this fact has not yet been fully explored and confirmed. By taking the antigenic RBD domain of the SARS-CoV-2 spike glycoprotein as a model building block, we investigated the production of nanoparticles and secretory microparticles out of the versions of recombinant RBD produced by bacteria (*Escherichia coli*), insect cells (Sf9), and two different mammalian cell lines (namely HEK 293F and Expi293F). Although both functional nanoparticles and secretory microparticles were effectively generated in all cases, the technological and biological idiosyncrasy of each type of cell factory impacted the biophysical properties of the products. Therefore, the selection of a protein biofabrication platform is not irrelevant but instead is a significant factor in the upstream pipeline of protein assembly into supramolecular, complex, and functional materials.

## 1. Introduction

**Figure 1:**
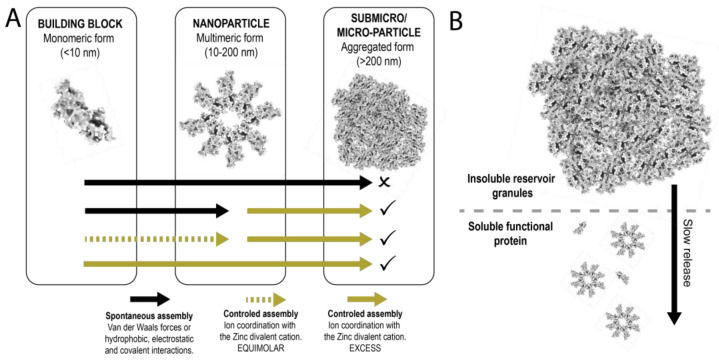
(**A**) In vitro formation of protein materials from monomeric (or protomeric) species. This occurs through oligomerization mediated by N-terminal cationic peptides plus C-terminal clustered histidine residues [35]. In most cases, assembly is spontaneous, as mediated by a set of cross-linking forces, such as those governing the formation of viral capsids. In others, the assembly must be assisted by the external addition of divalent cations [18]. A molar excess of divalent cations results in protein aggregation as microparticles with amyloidal architecture, which mimic secretory granules from the mammalian endocrine system [29]. (**B**) Under physiological conditions, these microparticles slowly disintegrate, releasing either monomers or nanoparticles [30,31]. Then, these materials act as depots for the slow in vivo administration of protein-based drugs. Upon subcutaneous administration [30], they mimic the behavior of functional, non-toxic amyloids, regarding the sustained release of the building block polypeptides [36,37,38,39,40,41].

Protein-based materials, that is, supramolecular structures with predefined properties [1,2,3,4,5,6], are of great interest in different clinical fields, including drug delivery, surgery, and regenerative medicine [7,8,9,10,11]. In contrast to synthetic materials, which pose concerns regarding organic [12], systemic [13], and environmental toxicity [14], the biodegradability and biocompatibility of proteins as materials make them highly suited for use in biological interfaces. The fabrication of protein materials is supported by the capability to engineer selected polypeptides that enable either spontaneous or inducible self-assembly. Under physiological conditions, such an arrangement should render stable cross-molecular interactions and, therefore, derive oligomers with regulatable levels of complexity. Since proteins show mechanical stability and biological activities, protein materials might combine both scaffolding and functional properties, being both fully biocompatible and a smart material [8,15]. Various engineering approaches allow the production of peptides and proteins that can self-assemble under the desired conditions. Apart from rational protein modification towards stabilization [16], the use of histidine (His)-rich segments as clustering tags enables coordination and molecular cross-linking with divalent cations from media [17,18], including those such as Ca^2+^, Mn^2+^, and Zn^2+^ that abound in biological tissues [6]. The His-based approach to protein assembly is universal, suited for any protein type [6,19,20], and useful for generating nanoscale or more complex microscale materials, depending on the molar ratio between cations and His residues [21] (Figure 1A). Therefore, tagging with the hexahistidine H6 or similar His-rich peptides is a simple approach that enables proteins of particular biomedical interest to be assembled as regular oligomers [6,17]. Protein nanoparticles obtained in this way, when empowered with ligands of cell-surface molecules, have proven extremely efficient vehicles for cell-targeted drug delivery in oncology [22,23,24], as well as being used in colorectal cancer [25], leukemia [23], lymphoma [26], melanoma [27], and head and neck [24] cancer. On the other hand, structurally related but more complex microparticles (Figure 1B) show promise as dynamic protein depots for the sustained delivery of protein drugs [28,29,30,31] or protein–drug nanoconjugates [32] in cancer therapies, of oligomeric nanobodies in type-2 diabetes [33], and of growth factors in regenerative medicine [34].

Two of the main appealing properties of materials generated by His-ion coordination are that (i) proteins might be derived from any source or cell factory, and (ii) any protein species is a potential building block for the generation of such oligomeric structures. In this scenario, recombinant proteins are ideal building blocks, since they are superior to synthetic peptides regarding their flexibility in design and engineering, because the absence of an upper length limit; the capacity to combine functional domains; and their industry-oriented, cost-effective, and scalable biological fabrication. In this context, many types of cell factories have been developed for recombinant protein production [15,42,43,44,45,46]. Apart from conventional bacterial and yeast species (e.g., *Escherichia coli*, *Bacillus subtilis*, *Saccharomyces cerevisiae,* and *Pichia pastoris*), mammalian cell lines, insect cells, baculovirus expression system, and other unconventional and emerging cell platforms can now be found in the catalogue of cell types suited for protein biofabrication [47]. While comparative studies regarding the productivity, post-translational modifications, functionality, and conformational quality of the resulting protein products are relatively common [48,49,50,51,52], the influence of the cell factory on the capability of the product to form structurally complex materials (that is, the architectonic quality of the building block) has so far been neglected. This information is of relevance regarding the potential industrial-scale production of these types of materials that be might derived from clinically interesting products developed at laboratory scale.

Reflecting the need for such an exploration and using an antigenic protein segment from SARS-CoV-2 as a model, we here explored the capability of the polypeptides resulting from three main protein production platforms (namely bacterial, insect, and mammalian cells) to form ion-mediated complex materials. The results generated here offer clues about the evaluation of particular properties of such platforms, supporting or advising against their preferential use for the effective production of functional protein materials.

## 2. Materials and Methods

### 2.1. Genetic Design

**Figure 2:**
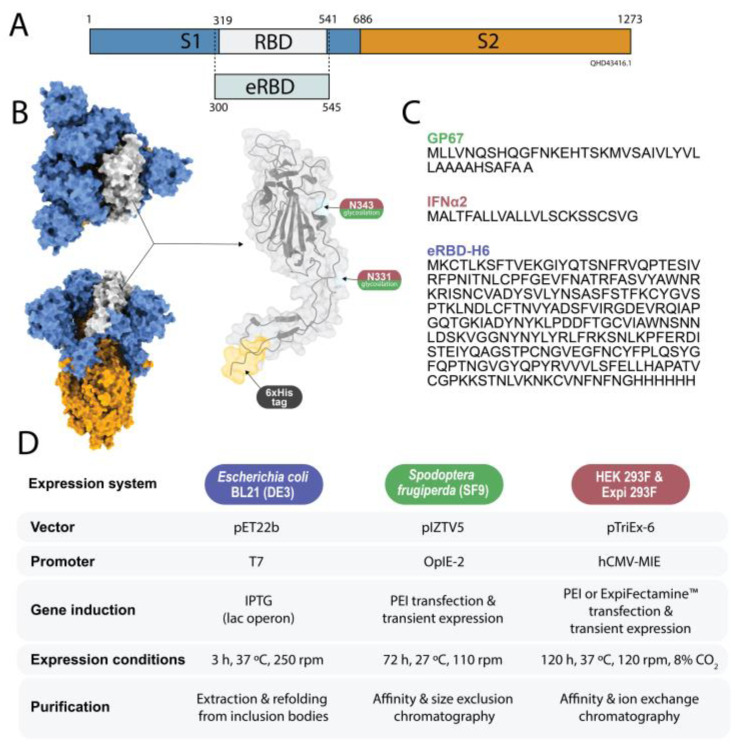
Protein design and production. (**A**) Selected segment of the subunit S1 of the SARS-CoV-2 spike glycoprotein used as model. The “e” in eRDB means extended, to indicate the inclusion of a cationic region from the viral capsid protein, beyond (at the N-terminus of) the consensus limits of the RDB segment. (**B**) Molecular model of the whole spike protein trimer (PDB: 6VXX). In grey, the selected eRDB domain, including the H6 tag and indicating the two glycosylation sites. (**C**) The explicit amino acid sequence of the eRBD construct and the used signal peptides are also depicted on the right. The C-terminal H6 peptide is included here. (**D**) The main features and working conditions of the production platforms used in this study are indicated.

An extended version (eRBD) of the RBD domain from SARS-CoV-2 (GeneBank: Accession No. QHD43416.1, [53]) was selected for comparative production in cell factories (Figure 2A). The encoding genes were codon optimized and supplied by GeneArt (Thermo Fisher, Waltham, MA, USA) subcloned into the vectors used for gene expression in the systems described below (Figure 2B). This viral domain was selected as a comparative protein because of its potential interest for further studies as a slow-release antigen. In addition, being a glycosylated stretch in the virus capsid, we expected that any effects that the glycosylating and non-glycosylating systems might impose on the performance and quality of the material might be then magnified and comparatively observed.

### 2.2. Protein Production in Bacteria

eRBDH6 was obtained in bacteria, by adapting a purification protocol described elsewhere [54], in which the recombinant protein was solubilized from inclusion bodies. The encoded protein was produced from the gene inserted into pET22b by *Escherichia coli* BL21 DE3 (Novagen-Merck, Darmstadt, Germany), growing at 37 °C during 3 h in Lysogeny Broth (LB) upon the addition of 0.5 mM isopropyl-β-d-1-tiogalactopyranoside (IPTG). Cells were then harvested using centrifugation (15 min at 5000× *g*) and resuspended in 50 mM Tris, pH 8.0, in the presence of protease inhibitors (cOmplete™ EDTA-Free, Roche, Basel, Switzerland, ref. 05056489001). Cells were then sonicated for 8 min at 40% amplitude (1 s ON, 4 s OFF) in a Branson digital sonifier (Branson, MO, USA) and the lysate was centrifuged at 8228× *g* for 30 min. The pelleted fraction was resuspended in cleaning buffer (1 M NaCl, 2 M Urea) and centrifuged again at 8228× *g* for 30 min. The washed pellet was then resuspended in solubilization buffer (1 mM EDTA, 15 mM DTT, 6 M Guanidine hydrochloride) and agitated for 90 min at room temperature, until the refolding step, at 4 °C. This was done by adding 15 volumes of refolding buffer (0.18 mM EDTA, 0.5 M L-arginine, 1.9 mM reduced glutathione, 0.9 mM oxidized glutathione, 2 M Urea in 20 mM phosphate buffer, pH 8.0) at a rate of 0.7 mL/min using a peristaltic pump.

A micro-dialysis assay was conducted with an array of 21 FDA-approved buffers, to determine the optimal storage conditions. Finally, the extracted and refolded protein was then two-fold concentrated in an Amicon Ultra-15 30 kDa centrifugal filter unit (Millipore, Burlington, MA, USA, ref. UFC9030) and dialyzed against a battery of buffers (Table A1), aiming to gently remove the chaotropic agent. Finally, the sample was centrifuged at 15,000× *g* for 15 min and the supernatant was stored at −80 °C.

### 2.3. Protein Production in Sf9 Cells

The eRBDH6 protein was produced by transiently transfecting Sf9 cells with the plasmid pIZT/V5-His encoding the gene sequence with the gp67 secretion signal peptide in the N-terminus, following a previously established protocol [55,56]. Briefly, Sf9 cells were grown in Insect-XPRESS medium (Lonza, Basel, Switzerland) supplemented with glutamine to a final concentration of 2 mM in disposable polycarbonate Erlenmeyer flasks (Thomson Optimum Growth Flasks, Sittingbourne, UK) and maintained under agitation in an orbital shaker at 110 rpm and 27 °C. Cells were subcultured every 2–3 days and maintained at a density of 1 × 10^5^ cells/mL. Transient transfection was performed using linear 25 kDa PEI (Polyethylenimine, Linear, MW 25000, Polysciences, Warrington, PA, USA, ref. 23966-100), which was prepared in water at a final concentration of 1 mg/mL, pH 7.0. Transfection was performed at high cell densities (15–20 × 10^6^ cells/mL) using a specific pDNA:PEI:vehicle ratio (pDNA at 1 pg/cell and PEI at 2 pg/cell), diluted in ultrapure water to a final volume corresponding to 10% of the cell culture volume. The pDNA:PEI mixture was vortexed for 3 × 3 seconds and incubated for 10 min at room temperature, then added dropwise to the cells, and incubated without agitation for 15 min. Then, cells were diluted with fresh medium to a final concentration of 4 × 10^6^ cells/mL and maintained under agitation for 72 h at 110 rpm and 27 °C.

The secreted protein was purified from the clarified supernatant of Sf9 culture by combining affinity chromatography and subsequent size exclusion chromatography. After centrifugation to remove cellular debris, the supernatant received 350 mM NaCl, 0.01% polysorbate 80 and the pH was adjusted to 7.2. The supernatant was centrifuged at 15,000× *g* for 20 min to remove all remaining cell debris. It was then applied to a HisTrap EXCEL column (Cytiva, Marlborough, MA, USA, ref. GE17-3712-06) with immobilized nickel for affinity chromatography (IMAC) in an ÄKTA system (Cytiva, Marlborough, MA, USA), which had previously been equilibrated with 20 mM Tris pH 7.2, 500 mM NaCl, 0.01% polysorbate 80. An additional washing step was performed with a polysorbate 80-free buffer. Elution was performed with an increasing linear gradient of imidazole in 20 mM Tris pH 7.2, 500 mM NaCl. The eluted specific peak was submitted to size exclusion chromatography in a HiLoad 16/600 Superdex 200 pg column (Cytiva, Marlborough, MA, USA, ref. GE28-9893-35), which had previously been equilibrated with 20 mM Tris pH 8.0, 150 mM NaCl. An isocratic elution was performed in the same buffer and the corresponding peak was collected. Protein was then concentrated using an Amicon Ultra-15 centrifugal filter unit 3 kDa (Millipore, Burlington, MA, USA, ref, UFC9003), centrifuged at 15,000× *g* for 30 min, and stored in aliquots at −80 °C for further use.

### 2.4. Protein Production in Mammalian Cells

The eRBDH6 protein was produced by transfection and transient gene expression in two different mammalian cell lines. The first was the suspension-adapted human embryonic kidney (HEK) cell line FreeStyle^TM^ 293-F (Invitrogen, Waltham, MA, USA, ref. R79007). These cells are adapted to grow in suspension in FreeStyle™ 293 Expression Medium (Gibco, Billings, MT, USA, ref. 12338018), a chemically defined, protein-free medium specifically developed to support the growth and transfection of FreeStyle™ 293-F cells under suspension culture conditions. Stock solution of linear 25 kDa PEI (Polyethylenimine, Linear, MW 25000, Polysciences, Warrington, PA, USA, ref. 23966-100) was prepared in water at a final concentration of 1 mg/mL, pH 7.0. The solution was sterilized using a 0.22 µm filter and separated in aliquots that were stored at −80 °C. The transfection conditions for the FreeStyle^TM^ 293-F cells were as previously set [57] and further optimized at 0.5 µg DNA/mL of culture and a ratio DNA:PEI of 1:3 (*w*/*w*). Valproic acid (VPA, Merck, Burlington, VT, USA, ref. P4543) was added to the cells (at 4 mM final concentration) 4 h post-transfection, in order to improve the recombinant protein expression.

The second system used Expi293™ cells and the related Expi293™ Expression System (Gibco, Billings, MT, USA, ref. A14635). Cells were transfected using the ExpiFectamine™ 293 reagent and kit, following the vendor’s protocol. To check protein production, 1 mL samples from transfected cell cultures were taken on different days post transfection and centrifuged (at 15,000× *g* for 10 min), and the supernatants and cell pellets were separated and stored at −20 °C, until being analyzed with SDS-PAGE. Cell pellets were resuspended in phosphate buffered saline (PBS) supplemented with a protease inhibitor cocktail (cOmplete, EDTA-free, Roche Life Sciences, Penzberg, Germany, ref. 05056489001) and kept at −20 °C, until cell lysis.

eRBDH6 was purified from supernatants of transfected cells using a combination of affinity capture followed by two polishing steps based on ion exchange chromatography. Affinity chromatography was performed in a HisTrap™ excel column (Cytiva, Marlborough, MA, USA, ref. 17-3712-06), prepacked with Ni Sepharose^®^ excel affinity media for capture and purification of the secreted His-tagged proteins, using IMAC. Columns were washed twice with buffer A (20 mM Tris-HCl pH 8.0, 500 mM NaCl) before the clarified supernatants were directly loaded into the column. After protein capture, the column was washed with buffer A, and then the proteins were eluted with a gradient of buffer A and buffer B (the same as buffer A but supplemented with 500 mM imidazole). Fractions were analyzed using SDS-PAGE and Western blot, and those containing the RBD protein were pooled and dialyzed against 12 mM sodium phosphate buffer pH 7.4. Then, the protein sample was refined using cation exchange chromatography with a strong cation exchanger HiTrap^®^ SP Fast Flow column (Cytiva, Marlborough, MA, USA, ref. 17-5054-01). In this case, and due to the theoretical pI (9.02) of the protein combined with the buffer pH (7.4), the recombinant product was expected to have a positive net charge and, therefore, it was expected to be captured by the negatively charged resin. A pool was loaded into the column and after capture, the column was washed with 12 mM sodium phosphate buffer at pH 7.4. Then proteins were eluted by increasing the ionic concentration with a gradient of phosphate buffer and elution buffer (the same as the phosphate buffer but supplemented with 500 mM NaCl). Fractions were analyzed using SDS-PAGE and Western blot, and those containing the RBD protein were again pooled and dialyzed against 12 mM sodium phosphate buffer pH 7.4. Finally, a final polishing was performed by loading the dialyzed sample into the strong anion exchanger Hi Trap Q Fast Flow (Cytiva, Marlborough, MA, USA, ref. 17-5053-01). In this case, only the negatively-charged contaminants were retained by the positively-charged resin.

### 2.5. Electrophoresis and Western Blot

To determine the protein expression, samples were analyzed with SDS-PAGE and further Western blot. For proteins expressed in mammalian and insect cells, the culture supernatants and total cell pellets resuspended in PBS were used to monitor the secreted and intracellular forms of the proteins, respectively. To detect recombinant proteins, SDS-PAGE was performed using TGX Stain-Free™ FastCast™ acrylamide 12% (Bio-Rad, Hercules, CA, USA, ref. 161-0185) and further visualization of the proteins with a ChemiDoc™ Touch Imaging System (Bio-Rad, Hercules, CA, USA). To visualize the immunoreactive bands in Western blots, anti-His mouse monoclonal antibodies (from Clontech, Mountain View, CA, USA, ref. 631212, or from GeneScript, Piscataway, NJ, USA, ref. A00186-100) were used as primary Abs. The samples to be quantitatively compared were run in the same gel and processed as a set. Densitometry analyses of the immunoreactive bands were performed with Image Lab™ software (version 5.2.1., Bio-Rad, Hercules, CA, USA).

### 2.6. Electron Microscopy

High-resolution electron microscopy images of soluble eRBDH6 protein were obtained with transmission electron microscopy (TEM) using a TEM Jeol 1400 (Jeol, Tokyo, Japan) with an operating voltage of 80 kV. This microscope was equipped with a Gatan Orius 8 9 SC200 CCD camera (Gatan Inc. Pleasanton, CA, USA), and representative images were captured from different fields at 20,000× and 25,000× magnifications. Sample preparation consisted in placing 10 µL droplets of protein sample (0.05–0.1 mg/mL) on top of glow-discharged 200-mesh carbon-coated copper grids (Electron Microscopy Sciences, Hatfield, PA, USA) for 1 min. Then, the excess liquid was blotted with a Whatman filter paper, and protein was negatively stained by placing the grid upside down over a 10 μL drop of 1% uranyl acetate (Polysciences Inc. Warrington, PA, USA) for 1 min. Excess liquid was blotted again and the grids were dried at room temperature, for at least 10 min, before image acquisition. On the other hand, high resolution images of cation-induced microparticles were obtained using field emission scanning electron microscopy (FESEM) using a FESEM Zeiss Merlin (Zeiss, Oberkochen, Germany) operating at 1 kV and equipped with a high-resolution secondary electron detector. To prepare the samples, 10 μL of each microparticle preparation (0.1 mg/mL) was directly deposited on silicon wafers (Ted Pella Inc. Redding, CA, USA) and left to dry at room temperature, before direct observation without a metallic coating.

### 2.7. Determination of Material Volume

The volume-size distribution of the protein materials was measured using dynamic light scattering (DLS) at 633 nm in a Zetasizer Nano ZS (Malvern Instruments, Malvern, United Kingdom) operating at a controlled temperature of 25 °C. For this, at least 50 µL of each protein solution (0.1–1 mg/mL) was measured in triplicate in low volume cuvettes. For the study of the particles released during physiological incubation conditions, particle determination was conducted by directly measuring the undiluted supernatants. Data were processed in ZS XPLORER software (version 2.0.1.1.).

### 2.8. Formation of Microparticles

Microparticle formation was induced with a molar excess of ionic Zn (regarding the number of histidine residues in the H6 tag) using an adaptation of a described procedure [19]. In brief, these protein clusters were constructed by mixing 100 µg of each soluble protein with an excess of ZnCl_2_ in a molar excess ratio of 1:300 (protein:cation) [31]. Precipitation of the soluble protein was carried out in duplicate in potassium-sodium phosphate-buffered saline at physiological pH 7.4 in a final volume of 100 µL and at a final protein concentration of 1 mg/mL. Reaction tubes were gently homogenized after mixing the components and left to react for 10 min. Then, samples were centrifuged at 15,000× *g* for 15 min, to separate the microgranules in the insoluble fraction from the unreacted soluble protein in the supernatant. The efficiency of aggregation for each protein was evaluated by comparing the soluble protein that remained after precipitation with the initial protein amount.

### 2.9. Analysis of Protein Release

For the release assay, a protocol was developed from previously described methods [39]. In brief, microparticles were incubated for 7 days at 37 °C in PBS (pH 7.4), without agitation. The released protein present in the soluble fraction of the mixture was fully harvested from the supernatant at days 1, 2, 3, 5, and 7 using centrifugation at 15,000× *g* for 10 min and quantified; and then fresh PBS was added to replace the subtracted volume. At the end of the incubation period, the remaining microparticles were exposed to EDTA at the same molar concentration as the divalent cation, to recover the Zn-chelated protein still available in the granules. The protein in the supernatant was quantified using the Bradford assay (Bio-Rad, Hercules, CA, USA). A Qubit™ Protein Assay Kit (Invitrogen, Waltham, MA, USA, ref. Q33211) was used for samples below the limit of detection. Release kinetics were studied using SDS-PAGE gels, where 10 µL of each supernatant at different incubation times was loaded, and the respective protein bands were quantified using Image Lab 5.2.1 software (Bio-Rad, Hercules, CA, USA). Size determination of the particles released into the supernatant was performed by DLS, where each detected peak was considered an independent population for data visualization purposes.

## 3. Results

### 3.1. Protein Production

New biocompatible, cost-effective, and regulatable drug delivery systems are required in different biomedical fields. The emergence of promising clinically oriented materials based on recombinant proteins, either nanoparticles or nanoparticle-secreting micro-granules, offers a promising approach to fulfilling such needs. In this context, we made a comparative evaluation of the three main expression systems used to generate efficient protein building blocks. These are intended for the construction of materials that are organized through the coordination of divalent cations [6] with overhanging His residues [17]. As far as we know, such a comparative analysis has to date been neglected in biomaterials science. This is an important issue, since the biological features of particular cell factories or associated downstream procedures might have influences over the self-assembly properties of the resulting building blocks and final materials. Therefore, we selected as a model protein building block the segment of the SARS-CoV-2 spike protein that includes the receptor-binding domain (RBD) of the virus (Figure 2A). This domain, which had previously been demonstrated as suited for recombinant production [53], was used as a common reference for comparison. The N-terminal end was slightly expanded over the consensual RBD, to incorporate a few cationic amino acids from the original viral sequence (Figure 2A). Cationic regions, as demonstrated previously, might favor assembly in hexahistidine tail (H6)-based oligomerization platforms [35]. Since the explored oligomerization mechanics are based on Zn-His coordination, a H6 tag was fused at the C-terminus of all constructs (Figure 2A–C). The insect and mammalian cell systems required additional signal peptides (Figure 2C and Section 2) to promote protein secretion, but both used segments that were expected to be cleaved upon biological protein production and secretion.

**Figure 3:**
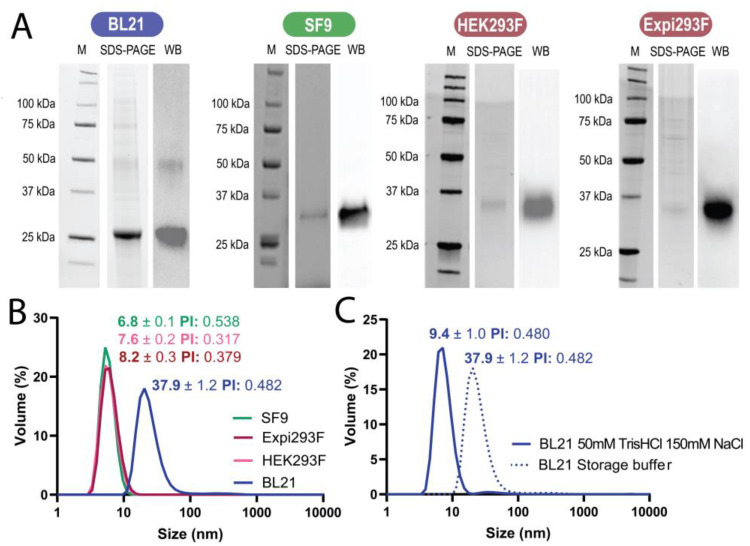
Comparative analysis of the protein products. (**A**) Electrophoretic analysis of the final protein products, including immunodetection using an anti-His antibody. (**B**) DLS plots of the purified proteins, to reveal their hydrodynamic size and polydispersity index (PI). (**C**) Disassembly of the bacterial product into protomers in low-salt buffer. In these conditions, the protein was unstable and tended to precipitate.

Upon platform-specific production and purification procedures (Figure 2D), the resulting polypeptides were observed as discrete bands, indicating a high proteolytic stability, with migration patterns close to the expected electrophoretic mobility (Figure 3A, Table 1). ProtParam (ExPASy) software predicted the molecular mass of the protein (the final product, once the signal peptides had been removed) as 28.6 kDa, and MALDI-TOF/TOF mass analysis indicated 28.6 kDa for the bacterial and 31.1 kDa for the insect cell products. The moderate purity of the mammalian cell product (Table 1), which was consistent in several production attempts, prevented fine analytical determination of the protein mass using MALDI-TOF. However, based on its electrophoretic mobility, this was estimated to be 35.5 +/− 0.3 and 35.8 +/− 0.3 kDa for the protein expressed in HEK 293F and Expi293F cells, respectively. The higher molecular masses in the eukaryotic products in comparison with the bacterial protein (Figure 3, Table 1) were attributed to glycosylation. The presence of much larger saccharide chains in the mammalian cell products than in the insect cell products [58] accounted for the lower mobility in the gels of the first group of species, thus indicating a higher predominant molecular size.

**Figure A1:**
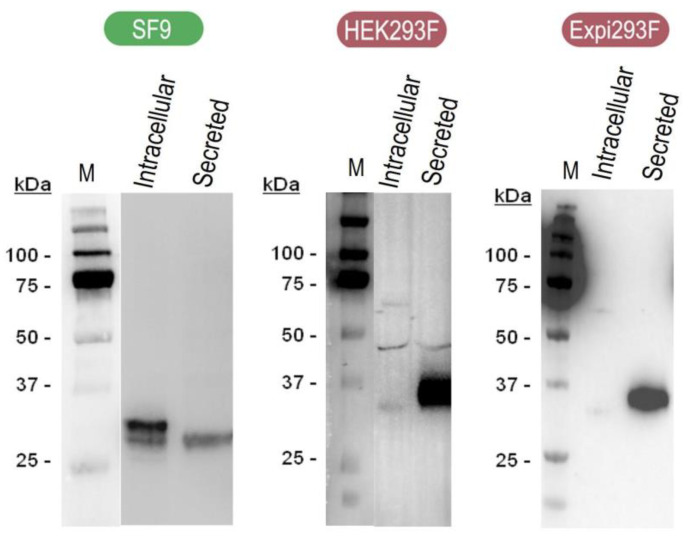
Electrophoretic analysis of intracellular and secreted eRBD produced in eukaryotic cells from Western blot immunodetected bands.

The processing of the signal peptide was clearly confirmed in the case of the insect cell protein product, which increased in electrophoretic mobility after secretion (Figure A1). The interpretation of the data from the mammalian cell production was less conclusive, since the combination of signal peptide removal and the presence of complex glycosylation rendered contradictory the contributions to the final protein size. However, the mobility-shifting pattern observed here (Figure A1) was fully in agreement with other studied cases, in which the signal peptide had efficiently been removed and in which the mass of the introduced glycan chains was higher than that of the cleaved signal peptide [59]. Partially glycosylated intracellular proteins would explain the lower molecular size compared with the fully extracellular proteins. In this context, the mass of the secreted form was observed as higher than that of the intracellular form. On the other hand, the yield of the bacterial products reached 40 mg/L, and that of the mammalian cell products was particularly modest (Table 1). The insect cells produced moderate protein levels, but still sufficient for a conformable characterization of the product.

### 3.2. Formation of Nanoparticles

**Figure 4:**
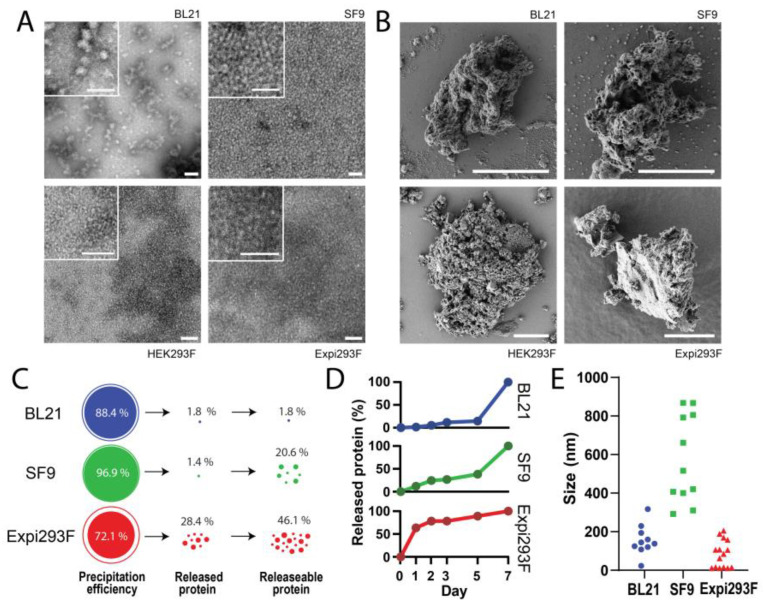
Comparative characterization of the protein materials. (**A**) TEM images of the purified protein samples used for the construction of nanoparticles. Only the bacterial products formed oligomers. Scale bar size: 50 nm. (**B**) FESEM images of the microparticles formed out of the materials shown in A. Scale bar size: 6 µm. (**C**) Precipitation efficiency of the soluble protein into microparticles, in % of the starting material. The fraction of released protein upon 7 days of incubation at 37 °C is indicated, as well as the total fraction of protein released to the soluble fraction from the granules, when forced by Zn chelation mediated by EDTA, at day 7. (**D**) Kinetics of protein release from the microscale granules, referring to the maximal amount of released protein at the end of the experiment. (**E**) Size of the materials released from microparticles in samples taken on days 1 and 3 of the 7-day release experiment.

The charge distribution of the designed protein (an N-terminal cationic region plus a C-terminal histidine tail) was expected to favor self-assembly as nanoscale oligomers [60,61,62], irrespective of the selected cell factory. A spontaneous formation of nanoparticles was confirmed with DLS in the case of the bacterial product (with a size of 37 nm) but not in the protein produced in eukaryotic cells, which remained monomeric and peaked at around 8 nm (Figure 3B). We confirmed that the protein peak of 37 nm corresponded to an oligomeric version of eRBD through the disassembly of the material in low-salt buffer, which rendered building blocks of around 9 nm (similar in size to the protein produced in eukaryotic cells, Figure 3C). This protein fraction tended to precipitate in the tested buffer (not shown), indicating that the assembled version was more stable than the plain building blocks. The formation of nanoparticles was confirmed by electron microscopy in the bacterial product but not from other sources (Figure 4A).

### 3.3. Formation and Disintegration of Microparticles

The purified protein samples recovered from each production (Figure 4A) were used as building blocks for the formation of microparticles, intending to act as secretory granules. All the protein samples rendered microparticles (Figure 4B) of a few microns in diameter and with irregular architecture. While FESEM images were taken, the HEK293F product was excluded from further explorations owing to its low yield (Table 1), which imposed analytical restrictions. The reasons for such a low yield, which was far below the common expectation of a few mg per liter that this system usually offers with comparable production protocols [63,64,65], are unclear and would need further analyses (and protocol adjustments, if necessary). The efficiency of ionic Zn in aggregating proteins into microparticles through coordination with His residues was generally high, ranging from 72 to 97%, as expected (Figure 4C). At this point, we were interested in the functionalities of these microparticles; that is, their ability to release protein in a sustained manner, for which they would have clinical value in drug delivery (Figure 4C,D). As noted (Figure 4C), less than 2% of the microparticle material was lost in the products derived from bacterial or insect cells. Almost 30% of the granular entities formed by the mammalian cell product spontaneously disintegrated in 7 days. While mechanical integrity is, of course, appealing regarding the handling and application of secretory granules in vivo [31], the bioavailability of the embedded protein is also a relevant factor regarding the clinical action of a drug. By chelating the Zn cations with EDTA at the end of the 7-day experiment, we observed that the total fraction of releasable protein was about 50% of the granular material in the mammalian cell products, with decreasing fractions for the insect cell and bacterial cell products (Figure 4C).

Furthermore, to assess the release kinetics of eRBDH6 in all three expression systems, soluble protein was quantified from all supernatant fractions relative to the amount released over a week-long period (Figure 4D). We observed that the bacterial and insect cell-derived microgranules released small amounts of soluble protein during the first few days, with increasing amounts released over time, until most of the released fraction was solubilized during the final days of the assay. In contrast, the mammalian material was preferentially disaggregated during the initial period of incubation, with a minimal release from day 2 onward. Briefly, in the mammalian platform, over 50% of the released protein was present by day 1, whereas it took over 5 days to achieve an equivalent proportion in the bacterial- and insect-derived materials.

Finally, to determine the geometry of the secreted protein and to determine if it occurred in the form of plain monomers of nanoparticulate materials, we analyzed the size of the soluble protein released in the incubation tubes during the 7-day incubation process. Interestingly, a range of products with different sizes were observed (Figure 4E, Table 1), which were clearly distinguishable when comparing protein sources. In any case, the occurrence of plain monomeric versions was not detected using DLS, but nanoparticles with bacterial and mammalian cell origins were indeed identified as soluble particles, generally under 200 nm. In the case of the protein produced in bacterial cells, the size range included that observed in the starting material resulting from purification (compare Table 1 and Figure 3B and Figure 4A,E). The microparticles resulting from insect cells instead disintegrated into larger soluble protein entities at submicron scale, between 300 and 900 nm approximately (Table 1, Figure 4C).

## 4. Discussion

The pharmacological treatment of most human diseases or chronic conditions requires a prolonged drug administration, to prevent undesired peaks and valleys in pharmacokinetics and to achieve constant, effective, and nontoxic levels [66,67,68]. Therefore, a variety of slow drug-delivery systems are under development, in which the active agent is embedded in a biocompatible matrix. Acting as dynamic depots, these allow a prolonged release of the carried drug [69,70,71,72]. Considering protein drugs, an interesting approach to achieving a fully biocompatible slow drug-delivery system is to package the protein drug in nontoxic, functional amyloids, which exploit the coordination properties of divalent cations and His residues [28,30]. By doing so, the resulting microparticles mimic the architecture and functioning of secretory granules from the mammalian endocrine system [36,37,40,41,73], in which peptide or protein hormones are clustered together by means of ionic Zn. In this approach, the protein drug itself is the holding system in which the building blocks keep attached together, in a reversible way and with the absence of external xenobiotic materials (apart from Zn at physiological quantities). Rather than protein release, the material self-disintegrates into its building blocks, namely functional monomers or oligomers. While this system has proven useful in different applications, including cancer and regenerative medicine [31,34], it remains poorly explored from a methodological point of view. In the context of such need, we explored how the cell factory used for protein production might determine or impact the structural and functional performance of the secretory granules. To date, bacteria have been the only system used for protein production intended for His-based clustering [20,28,30]. While these types of cell are excellent biofactories for proteins, specific needs related to post-translational modifications might advise the exploration of other protein sources, a possibility that had not previously been investigated.

Apart from the system-dependent differences found in the productivity and final purity of the model glycosylated RBD from SARS-CoV-2 (Table 1), the same polypeptide was obtained from the four production systems (Figure 1) and was found to efficiently produce microparticles (Figure 4B) with relatively high efficiency (Figure 4C) upon Zn-mediated clustering. In addition, these materials act as dynamic depots. In an in vitro experimental setting for the analysis of protein leakage, all of them released soluble protein species (Figure 4C), with the mammalian cell product being the fastest regarding disintegration (Figure 4D). Important differences were, however, observed in the spontaneous tendency to oligomerize, which is often observed in engineered, H6-tagged polypeptides upon purification [18]. In this regard, the bacterial product showed a clear tendency to form nanoparticles, which was not observed in the rest of the tested systems (Figure 3B and Figure 4A). However, when analyzing the size of the released protein materials during the disintegration of the secretory microparticles, nanostructured entities were observed in all cases (Figure 4E). Why the materials formed by insect cell-derived products rendered larger protein complexes is unclear, but this was a systematic and consistent observation in all the tested samples (Figure 4E). This fact, together with the differential dynamics of protein release, suggests that the process was affected by the glycosylation pattern, as was confirmed in both mammalian and insect cell products, (Figure A1) in the cross-interactivity between the polypeptides, and also in the mechanical stability of the resulting material. Indeed, glycosylation has shown a great influence on the interactivity of target polypeptides in a variety of biological contexts [74,75,76] and also on their structural stability [77,78]. Of course, additional exploration is needed to confirm this possibility regarding the construction of dynamic depot materials such as those generated here. In any case, all tested products showed valuable properties as building blocks for protein depots, and the differential functional profile observed among them could be of interest for their adaptation to particular clinical needs, regarding the optimal drug release rate.

## 5. Conclusions

Functional secretory granules formed by an extended recombinant version of the SARS-CoV-2 RBD were successfully fabricated in vitro. For this purpose, recombinant RBD versions used as building blocks were produced in bacterial and insect cells and in two mammalian cell lines. Upstream, midstream, and downstream production phases were optimized for each of these protein products, to achieve the maximal recovery yields and purity. Despite the inherent variability in all these processes, the four products were suitable for producing functional secretory granules at the microscale, which during their spontaneous disintegration released the formed protein in a sustained manner. This observation validated the concept that artificial secretory granules based on recombinant proteins can be formed irrespective of the cell factory used as a protein source. Nonetheless, important differences were found in the spontaneous assembly of nanoparticles upon recombinant protein production and purification, and the bacterial cell factory system clearly favored oligomerization. Moreover, the disintegration kinetics was slower in the bacterial and insect cell products compared to the mammalian cell products, and the protein secreted from bacteria- and mammalian-cell-derived materials adopted a conventional oligomeric disposition at nanoscale size. The occurrence of small multimers is appealing from a therapeutic point of view, compared to submicron particles, if internalization into target cells is envisaged. All these facts, taken together with the highest recovery yields and cost-effectiveness, indicates that, in absence of other biological or functional considerations, the bacterial system is the ideal source of components of homo-oligomeric nanoparticles and secretory microparticles intended for slow protein drug release.

## Figures and Tables

**Table 1:** Protein yield and purity of the tested protein production platforms.

Host	Productivity (mg/L)	Purity (%)	Molecular Mass (kDa)	Proteolysis (Y/N)	Spontaneous Formation of Nanoparticles (Y/N; size, nm)	Formation of Microparticles (Y/N)	Release of Nanoparticles (Y/N; Size, nm)
*E. coli*	40	>90	28.6	N	Y (37.9)	Y	Y (23.3–317.4)
sf9	1.50–1.64	>98	31.1	N	N	Y	Y (291.9–868.0)
HEK293F	0.15	<60 ^ii^	35.5 (33.1 ^i^)	N	N	Y	Y (ND)
Expi293	0.38	<60 ^ii^	35.8 (32.6 ^i^)	N	N	Y	Y (11.1–205.8)

**^i^**. intracellular ^ii^. Values were typically between 50 and 55%. ND—not determined.

## Data Availability

Data are available from https://doi.org/10.34810/data685, accessed on 5 April 2023.

## Appendix A

**Table A1:** Battery of buffers used to gradually remove urea in the final dialysis step for protein purification from *E. coli*.

	Refolding Buffer	Step 1	Step 2	Step 3	Step 4	Storage Buffer
Phosphate buffer pH 8	20 mM	20 mM	20 mM	20 mM	20 mM	20 mM
EDTA	0.18 mM	-	-	-	-	-
Gluthathione red.	1.9 mM	-	-	-	-	-
Gluthatione ox	0.9 mM	-	-	-	-	-
L-Arginine	0.5 M	-	-	-	75 mM	75 mM
Urea	2 M	1 M	0.5 M	0.02 M	-	-
Sucrose	-	-	292 mM	292 mM	233 mM	233 mM
Polysorbate 20	-	-	0.25 mM	0.25 mM	0.25 mM	-
